# Planning trajectory for UAVs using the self-organizing migrating algorithm

**DOI:** 10.1371/journal.pone.0327016

**Published:** 2025-07-07

**Authors:** Quoc Bao Diep, Thanh-Cong Truong, Ivan Zelinka

**Affiliations:** 1 Faculty of Mechanical-Electrical and Computer Engineering, School of Technology, Van Lang University, Ho Chi Minh City, Vietnam; 2 Faculty of Information Technology, University of Finance-Marketing, Ho Chi Minh City, Vietnam; 3 Department of Computer Science, Faculty of Electrical Engineering and Computer Science, VŠB-Technical University of Ostrava, Ostrava-Poruba, Czech Republic; 4 IT4Innovations, Quantum Computing Lab, VŠB-Technical University of Ostrava, Ostrava-Poruba, Czech Republic; Southwest Jiaotong University, CHINA

## Abstract

Ensuring efficient and safe trajectory planning for UAVs in complex and dynamic environments is a critical challenge, especially for UAVs that are increasingly deployed in applications like environmental monitoring, disaster management, and surveillance. The primary complications in the safe control of UAVs include real-time obstacle avoidance, adaptation to unpredictable environmental changes, and coordination among multiple UAVs to prevent collisions. This paper addresses these challenges by proposing a novel approach for UAV trajectory planning that integrates obstacle avoidance and target acquisition. We introduce a new cost function designed to minimize the distance to the target while maximizing the distance from obstacles, effectively balancing these competing objectives to ensure safety and efficiency. To optimize this cost function, we employ the self-organizing migrating algorithm, a swarm intelligence algorithm inspired by the cooperative and competitive behaviors observed in natural organisms. Our method enables UAVs to autonomously generate safe and efficient paths in real-time, adapt to dynamic changes, and scale to large swarms without relying on centralized control. Simulation results across three scenarios-including a complex environment with ten UAVs and multiple obstacles-demonstrate the effectiveness of our approach. The UAVs successfully reach their targets while avoiding collisions, confirming the reliability and robustness of the proposed method. This work contributes to advancing autonomous UAV operations by providing a scalable and adaptable solution for trajectory planning in challenging environments.

## 1 Introduction

The control of unmanned aerial vehicles (UAVs) has attracted the attention of researchers due to its wide application in many important fields, from environmental monitoring [1], to smart agriculture [2], search and rescue [3], disaster management [4] and logistics [5]. One of the primary tasks of UAVs is to plan effective flight trajectories and avoid unknown obstacles, ensuring that these UAVs can safely move to targets in unknown complex environments without any collisions. However, the sudden appearance and movement of dynamic obstacles, other UAVs, as well as unpredictable changes in the environment pose a significant challenge, which requires solutions that can solve the problem in real-time alongside other strict criteria such as minimum time, shortest distance or the minor energy consumption.

Traditional methods, which rely on predefined algorithms and operate in specific environments, often require human intervention to deal with such real-life situations. To address these challenges, swarm intelligence (SI) algorithms have emerged as a potential technique by leveraging the cooperative-competitive behavior of a group of individuals to perform intelligent action [6,7].

Several SI algorithms have been proposed and applied for path planning and obstacle avoidance for UAVs. For example, the Ant Colony Optimization (ACO) mimics the ants foraging behavior to find an optimal path [8]. The Particle Swarm Optimization (PSO) mimics the movement of particles to find the best trajectory [9]. Genetic Algorithm (GA) uses evolutionary principles to evolve initial solutions and optimize the flight paths of UAVs [10], and other evolutionary algorithms [11]. Another robust algorithm investigated in this study is the self-organizing migrating algorithm (SOMA) [12–14]. These algorithms provide different trade-offs between exploration and exploitation processes, between criteria such as accuracy and convergence speed, keeping a diversity of solutions that help users properly solve problems.

The SOMA is inspired by the competitive-cooperative behavior observed in animals and some insect communities. These natural systems can solve complex problems through simple behaviors of locally communicating individuals or interactions between individuals and the living environment. SOMA aims to simulate these principles in artificial systems, allowing groups of UAVs to explore and navigate their movements intelligently to avoid static and dynamic obstacles and catch their target.

One of the SOMA’s main advantages in such a UAV problem is its ability to handle dynamic and uncertain environments [15]. As they operate in real scenarios, the SOMA allows them to continuously generate their trajectory based on current dynamic conditions, ensuring safe and efficient movement when encountering dynamic obstacles or environmental changes.

Furthermore, the SOMA provides scalability and robustness in large-scale scenarios [16]. As the number of UAVs increases, the complexity of the problem increases exponentially [17]. Traditional methods can face slowness in handling the sizeable computational load of optimizing flight paths for many UAVs [18]. In contrast, the SOMA distributes operations across the swarm, allowing independent concurrent operations and decentralized control, as typical for swarm systems. This decentralization makes the SOMA suitable for large-scale applications, where a group of UAVs move and coordinate in a synchronized manner.

In this article, we propose methods to build the cost function for the problem of moving, avoiding obstacles, catching targets, and applying SOMA to plan real-time trajectories. Obstacle avoidance capabilities and algorithm adaptation for navigating UAVs in unknown environments are investigated. In particular, the central aspects of trajectory planning problems, including dynamic obstacle avoidance, target capture, stability assurance, and scalability for large-scale scenarios, are thoroughly investigated.

The key contributions and innovations of using SOMA for UAV trajectory planning and obstacle avoidance are as follows:

- **Novel cost function design**: We have proposed a new method to construct a cost function tailored for UAV path planning in complex environments. This cost function uniquely combines the objective of minimizing the distance to the target while maximizing the distance from obstacles, formulated using exponential functions to effectively balance these competing goals. This design ensures that UAVs can navigate efficiently toward their targets while safely avoiding both static and dynamic obstacles.
- **First application of SOMA to UAV trajectory planning**: While SOMA has been successfully applied in various optimization problems, to the best of our knowledge, its application to UAV trajectory planning with obstacle avoidance is novel. Our work extends the use of SOMA to this new domain, showcasing its suitability for real-time path planning in dynamic environments.
- **Enhanced handling of dynamic and uncertain environments**: SOMA’s mechanism inherently balances exploration and exploitation during the search process. This characteristic allows UAVs to adapt to changes in the environment on-the-fly, making it more effective in handling dynamic obstacles and unpredictable environmental conditions compared to traditional algorithms like PSO or ACO, which may converge prematurely or require reinitialization in changing environments.
- **Scalability and decentralization**: Our approach leverages SOMA’s decentralized computation and control, making it highly scalable for large-scale UAV systems. Unlike centralized methods that can become computationally intensive with an increasing number of UAVs, SOMA distributes the computational load across the swarm. This enables efficient operation even as the number of UAVs and obstacles increases, without significant degradation in performance.
- **Improved obstacle avoidance capabilities**: The cooperative-competitive behavior modeled by SOMA allows UAVs to effectively coordinate their movements to avoid both static obstacles and dynamic obstacles (other UAVs). This reduces the likelihood of collisions and enhances the safety and reliability of the UAV swarm operations.

The remainder of the article is organized as follows: Sect 2 provides an overview of related work in avoiding obstacles and catching target problems. Sect 3 presents the formulation of the problem and describes the self-organizing migrating algorithm and its applicability in the UAV trajectory planning problem. Sect 4 outlines the experimental setup, including the simulation environment and evaluation metrics used in the study. Sect 5 presents the experimental results and analyzes the effectiveness of the proposed method based on the gathered data. Finally, Sect 6 concludes the article by summarizing the main findings, discussing the contributions, and outlining potential directions for future research.

## 2 Related work

In recent years, many studies have been on using swarm algorithms in path planning and obstacle avoidance for UAVs. These research works have investigated and compared the effectiveness of different swarm intelligence algorithms in natural environments.

The article by Chen *et al*. [19] investigated the challenge of achieving rapid convergence in large-scale UAV swarms, a crucial factor for mission success. They proposed a novel fractional-order flocking algorithm (FOFA) that utilizes historical information during flight, effectively improving convergence rates compared to traditional integer-order flocking algorithms. The FOFA incorporates fractional calculus into the velocity updating process, allowing UAVs to consider past information in their decision-making. The authors theoretically analyzed the convergence of FOFA, deriving sufficient conditions for stability using graph theory. Simulation results demonstrated the superiority of FOFA, showing significant reductions in convergence time for swarms of varying scales (N = 50, 100, 200, 300, and 400) compared to traditional methods. Notably, the convergence rate was found to be strongly correlated with the fractional order of FOFA, with smaller fractional orders (indicating greater consideration of historical information) leading to faster convergence. The study also validated FOFA’s performance in wind environments and in multi-layer swarms with leader-follower structures. The authors concluded that FOFA offers a promising approach for controlling large-scale UAV swarms, particularly in scenarios where rapid convergence is essential. However, the study acknowledged that further research is needed to explore the algorithm’s applicability in more complex and realistic scenarios, such as navigation and obstacle avoidance.

Deng *et al*. [20] developed an autonomous sortie scheduling model for carrier aircraft fleets under towing mode, specifically addressing the challenges introduced by the inclusion of unmanned aerial vehicles (UAVs) on aircraft carriers. The primary objective was to solve the spatiotemporal coordination problem involving resource allocation and collision avoidance among heterogeneous dispatch entities, such as UAVs and towing tractors, on the confined flight deck. By modelling the sortie scheduling problem as a hybrid flow-shop scheduling problem (HFSP), they abstracted eight key processing procedures relevant to UAV towing operations. Tractors, preparing spots, catapults, and launching operations were virtualized as machines within the HFSP framework.

To efficiently solve the formulated HFSP, which is essentially a combinatorial optimization problem with tightly coupled constraints, they developed a chaos-initialized genetic algorithm (CiGA) with specially designed operators. The CiGA enhanced population diversity and improved convergence efficiency, outperforming standard genetic algorithms (GA) and particle swarm optimization (PSO). The proposed method ensured collision avoidance and effective resource allocation among heterogeneous dispatch entities, crucial for UAV operations on the flight deck. Simulation results demonstrated significant improvements; for instance, with eight aircraft, the total sortie duration was reduced from 436.3 seconds (using traditional constraints) to 378.3 seconds with the proposed model, showcasing enhanced UAV sortie efficiency. The research provided a foundational approach that could be extended by incorporating dynamic planning to handle uncertainties in UAV landing times and exploring advanced optimization methods to improve computational efficiency and scalability.

Wang *et al*. [21] developed a resilient multi-objective mission planning framework for UAV formations, integrating both task pre-assignment and re-assignment modules to enhance mission reliability. The primary objective was to address mission execution reliability by introducing probability constraints to ensure a minimum mission success rate and efficiently handling unexpected events through real-time task re-assignment. They formulated the task pre-assignment as a multi-objective optimization problem, aiming to maximize the expected value of successfully attacked targets while minimizing combat costs, including the total flight range and the value of destroyed UAVs. To solve this complex problem, they proposed an improved genetic algorithm with a multi-population mechanism and specifically designed evolutionary operators, termed the Multi-Population Multi-Objective Evolutionary Algorithm (MPMOEA). This algorithm utilized a custom chromosome encoding method and included tailored crossover and mutation operators to maintain feasible solutions respecting the imposed constraints.

In the task re-assignment phase, they analyzed potential trigger events such as UAV destruction or task execution failures and proposed a real-time Contract Net Protocol-based algorithm (RTCNP-CTRAP) to address these emergencies. The algorithm adapted the multi-objective optimization into a single-objective optimization using a weighted sum method to maintain consistent combat intentions during real-time adjustments. Their results demonstrated that MPMOEA outperformed traditional single-population algorithms, achieving better solutions and faster convergence. For instance, in a scenario with 6 UAVs and 11 targets, MPMOEA required fewer computations per iteration (86,400) compared to SPMOEA (259,200), leading to reduced CPU runtime. Additionally, the RTCNP-CTRAP achieved an average CPU runtime of approximately 0.1016 milliseconds for task re-assignment, showcasing its real-time capability.

Ye Lin *et al*. [22] addressed the problem of UAV swarm obstacle avoidance in multi-narrow type obstacle scenarios, where traditional methods struggle due to congestion and competitive allocation issues. They proposed a dual-game based Flocking (DGF) algorithm, which divides the flight process into two stages: maintaining the swarm’s flight state when no obstacles are present and implementing matching separation and motion state switching using a dual-game strategy when encountering multi-narrow type obstacles. The DGF algorithm leverages a game theory approach to determine the motion states of each UAV, ensuring orderly passage through the obstacles. Simulation results for both small-scale (N = 8) and large-scale (N = 28) UAV swarms demonstrated the effectiveness of the DGF algorithm in achieving smooth obstacle avoidance while preventing collisions. The algorithm successfully navigated the UAV swarm through multiple small holes, achieving a passage time of 1200 time steps with a decision distance of 40 m. The study also highlighted the algorithm’s ability to dynamically adjust UAV motion states based on environmental conditions, maintaining a quasi α-lattice structure and ensuring safe flight. However, the authors acknowledged the need for further research to explore the algorithm’s performance in more complex and dynamic environments with varying obstacle types and densities.

Aparajita Chowdhury and Debashis De [23] addressed the challenge of 3D path planning for UAVs in dynamic environments, aiming to find optimal collision-free trajectories while minimizing energy consumption. They proposed a novel approach called RGSO-UAV, which leverages the Reverse Glowworm Swarm Optimization (RGSO) algorithm to generate efficient paths. The RGSO-UAV algorithm considers a cost function that incorporates path length, altitude, fuel consumption, and power consumption, and uses a grid-based representation of the environment. The algorithm iteratively selects neighboring grid points with the lowest cost value, avoiding static and dynamic obstacles. The initial path is further optimized and smoothed to generate a tuned path. Simulation results showed that RGSO-UAV outperformed existing algorithms like GWO, GSO, PIOFOA, and PPPIO, achieving a 49%–107% reduction in cost function value and a 2%–61% reduction in execution time. However, the study acknowledged limitations in dealing with dynamic obstacles with random velocities and frequent changes in direction. The authors proposed further research to address these limitations and explore the application of RGSO-UAV in more complex and realistic scenarios.

Chen *et al*. [24] addressed the coverage path planning problem for heterogeneous UAVs in cooperative search systems with multiple separated regions, aiming to minimize total task completion time while efficiently visiting all regions. They proposed a linear programming-based formulation to determine exact flight paths and developed an Ant Colony System (ACS)-based heuristic algorithm consisting of region allocation and order optimization phases. Experimental evaluations compared the ACS-based algorithm with existing methods, including a Mixed Integer Linear Programming (MILP) formulation, a genetic algorithm, the Shortest Distance First (SDF) algorithm, and two spatial-temporal clustering-based algorithms (STCA-NA and STCA-NE). The ACS-based approach achieved lower task completion times than the GA, SDF, STCA-NA, and STCA-NE algorithms (e.g., for 50 regions, task completion time was 145.5 minutes compared to 150.8 minutes for GA and 162.6 minutes for SDF), and demonstrated better deviation ratios (e.g., 1.21% for ACS vs. 4.27% for GA when covering 50 regions). However, higher execution times compared to simpler heuristics (e.g., 125.38 seconds for ACS vs. 0.041 seconds for SDF when covering 20 regions), and the assumption that each region requires only single coverage, which may not align with real-world scenarios requiring multiple visits or continuous monitoring.

The paper [25] addressed the coverage path planning problem for autonomous heterogeneous UAVs operating over multiple separated regions, aiming to find efficient flight paths for UAVs to cover all regions of interest in the shortest possible time. They proposed an exact Mixed Integer Linear Programming (MILP) formulation to generate optimal flight paths but acknowledged its computational complexity for large instances. To overcome this, they developed a Spatial-Temporal Clustering-based Algorithm (STCA), inspired by the Clustering by Fast Search and Find of Density Peaks (CFSFDP) method. The STCA algorithm classified regions into clusters based on their densities calculated via relative distances and assigned clusters to UAVs. Experimental results demonstrated that the STCA algorithm could efficiently provide feasible flight paths, with the largest execution time being 0.93 seconds for up to 100 regions. However, the algorithm focused on offline planning with the assumption of fully known environments and did not address dynamic changes or uncertainties, limiting its applicability in real-world scenarios, and the time complexity of $\text{O}({\text{m}}^{2})$ could hinder scalability for larger problem instances.

The study [26] addressed the automatic path planning problem for heterogeneous UAVs, aiming to generate efficient flight paths that enable UAVs to cover all regions of interest while minimizing the total mission time. They modeled the heterogeneous UAVs and formulated the path planning task as a multi-constraint optimization problem, which they initially solved using a linear programming approach. Recognizing the computational complexity of exact solutions, they proposed an Adaptive Clustering-Based Algorithm (ACBA) inspired by density-based clustering analysis and Symbiotic Organisms Search (SOS) optimization. The ACBA involved evaluating UAV capabilities, clustering regions based on densities computed via relative distances and scan areas, and optimizing the visiting orders of regions using the SOS-based strategy. The experimental results demonstrated that the ACBA outperformed several existing methods, such as HETRF, HETRF-GA, APPA, and STCA-NA, in terms of task completion time. For instance, when covering 180 regions, the ACBA achieved a task completion time of 319.1 minutes, which was 7.5% less than HETRF and up to 5.1% less than STCA-NA.

The research [27] addressed the cooperative behaviour control problem of multiple UAVs in dynamic environments, aiming to design an algorithm that quickly produces safe and effective behaviour decisions for each UAV to achieve group missions. They modelled the motion and coordination of UAVs, analyzing collision avoidance, motion state updates, and task execution constraints, and abstracted the cooperative behaviour control as a multi-constraint decision-making problem. To solve this, they proposed a multi-agent reinforcement learning algorithm enhanced with a global-and-local attention mechanism, inspired by the human-learning process where attention is focused on important parts of data. The proposed algorithm, based on the Multi-Agent Deep Deterministic Policy Gradient (MADDPG) framework, allowed UAVs to cooperatively control behaviours and achieve coordination more effectively. Experimental results in a multi-agent particle environment demonstrated that their approach outperformed baseline algorithms (MADDPG, A3C, and DDPG) in mean reward, training time, and coordination effect. For example, in the predator-prey environment, their approach achieved a mean reward of 27.44, which was 1.15, 1.70, and 2.02 times larger than those of MADDPG, A3C, and DDPG, respectively.

Mohammad Kamrul Hasan *et al*. [28] addressed the challenge of UAV-to-UAV (UAV2UAV) interaction in swarm intelligence enabled decentralized networks, specifically focusing on the accurate determination of 3D relative positions of UAVs during flight. They identified a gap in existing research, which often lacked a comprehensive approach for tracking relative directions (left, right, up, down, back, forward) in 3D space, leading to path planning errors and potential network disruptions. To address this, they proposed a novel UAV2UAV-RDI model that incorporates six relative directions, utilizing two algorithms: UAV Relative Direction Identification (UAV-RDI) and UAV Relative Direction Tracking (UAV-RDT). The model uses evolutionary computation to track the relative 3D position of one UAV (tracking UAV) from the perspective of another UAV (handoff UAV). The authors demonstrated the model’s ability to track 24 different 3D positions of the tracking UAV, outperforming existing models that were limited to fewer directions or specific scenarios. The study highlights the importance of relative direction-based interaction for accurate 3D path planning and network stability in UAV-based SI systems. However, the model has limitations in considering angular aspects and the initial selection of UAVs for interaction. The authors suggest further research to address these limitations and explore the potential of the model in blockchain-based decentralized UAV networks.

Feifei Zhao *et al*. [29] addressed the challenge of collision avoidance in drone swarms, aiming to develop a self-organizing, decentralized approach inspired by the cooperative behavior of biological swarms. They proposed a model that utilizes reward-modulated spiking neural networks (RSNN) for online learning and decision-making by each individual drone. The RSNNs are trained independently based on local observations, enabling the swarm to emerge with autonomous collision avoidance capabilities in bounded spaces. Simulation and real-world experiments with up to five drones demonstrated the effectiveness of the proposed method, achieving a significant reduction in collisions compared to artificial neural network-based methods like LSTM and FCN. For example, in simulations with 25 drones and a collision threshold of 70, the RSNN-based approach resulted in an average of only eight collisions, whereas LSTM and FCN exhibited more frequent collisions. The study also highlighted the faster convergence and stability of the RSNN-based approach compared to ANN-based methods. However, the authors acknowledged that the model’s performance might be affected by inaccurate positioning in real-world scenarios with a large number of drones. They suggested future research to address this limitation and explore more complex cooperative and competitive decision-making tasks within drone swarms.

Kun Li *et al*. [30] addressed the challenge of multi-UAV task assignment and path planning for transmission line inspection in complex mountain environments, specifically considering the influence of multiple wind fields. They proposed a novel multi-mechanism swarm optimization approach that combines the strengths of different algorithms. For task assignment, they introduced a BACOHBA algorithm, integrating a bidirectional ant colony optimization with a discrete honey badger algorithm. This approach aimed to improve the global and local search capabilities, resulting in more efficient and accurate task allocation. For path planning, they proposed an HBAFOA algorithm, combining the honey badger algorithm with the fruit fly optimization algorithm to address the issue of excessive step size and out-of-bounds situations. Simulation experiments using public datasets (eil76 and rat575) and a custom mountain environment with multiple wind fields demonstrated the effectiveness of the proposed methods. The BACOHBA algorithm outperformed other task assignment algorithms like BAS-GA, OPA, and A-QCDPSO in terms of solution accuracy and convergence speed. Similarly, HBAFOA showed superior performance compared to ACO+AGWO, ACO+APSO, ACO+HBA, ACO+IBA, ACO+ORPFOA, and ACO+PIOFOA in path planning, achieving a significant reduction in cost (e.g., 7307.4 for BACOHBA+HBAFOA vs. 8516.1 for ACO+AGWO). The study highlighted the importance of considering wind field influences in multi-UAV inspection tasks and demonstrated the benefits of combining multiple optimization mechanisms for improved performance. However, the authors acknowledged that further research is needed to reduce the running time of the algorithms and refine the wind field quantification scheme for more accurate path planning.

Yanbiao Niu *et al*. [31] addressed the challenge of moving target search using UAVs, aiming to improve the efficiency and accuracy of finding dynamic targets in complex environments. They proposed an improved Sand Cat Swarm Optimization algorithm (ISCSO) that incorporates several strategies to enhance the algorithm’s performance. These strategies included a motion-encoding mechanism to represent search paths as a sequence of UAV movements, an elite pooling strategy to store and utilize high-quality solutions, and an adaptive T-distribution to balance exploration and exploitation. The ISCSO algorithm was tested in nine different search scenarios, demonstrating its superior performance in terms of cumulative probability and search time compared to other metaheuristic algorithms like seagull optimization algorithm (SOA), sand cat swarm optimization algorithm (SCSO), and improved wolf optimization algorithm (IGWO). For example, in scenario 1, ISCSO achieved a best cumulative probability of 0.1886 compared to 0.1258 for SOA, 0.1663 for SCSO, and 0.6389 for IGWO. The study highlighted the effectiveness of ISCSO in escaping local optima and finding high-probability target regions. However, the authors acknowledged that the model focused on single-UAV search and suggested future research to explore multi-UAV collaborative search for dynamic targets.

Chen Huang *et al*. [32] addressed the challenge of UAV path planning in complex 3D environments with multiple threats, aiming to improve the performance of traditional Particle Swarm Optimization (PSO) algorithms. They proposed a novel ACVDEPSO algorithm that integrates adaptive parameter adjustment, cylinder vector representation, and a differential evolution operator. The ACVDEPSO algorithm adaptively adjusts the inertia weight and acceleration coefficients based on the fitness values of particles and time, enhancing the search efficiency and preventing premature convergence. The cylinder vector representation facilitates more accurate and efficient path search in 3D space. Furthermore, the differential evolution operator, based on the global best solutions from previous iterations, helps the algorithm escape local optima. Simulation experiments using real DEM maps demonstrated the superiority of ACVDEPSO over other PSO variants. For example, in scenario 3, ACVDEPSO achieved a mean fitness value of 5602 compared to 5826 for PSO, 5916 for PSO-LDIW, and 6138 for θ-PSO. The ACVDEPSO algorithm also exhibited faster convergence speeds. However, the authors acknowledged that future research should focus on multi-UAV path planning and explore the impact of different parameters on algorithm performance.

Although there has been extensive research on the use of swarm algorithms in path planning and obstacle avoidance for UAVs, there still exist some challenges and limitations that need to be addressed. One of the challenges is the computational complexity and execution time of the algorithms, especially as the number of UAVs increases and the environment becomes more complex. Additionally, ensuring safe movement, obstacle avoidance, and successful target acquisition within a UAV swarm is a crucial issue that needs to be considered.

The following section will present how to construct the cost function for trajectory planning and obstacle avoidance in UAVs.

## 3 The proposed methodology

In this section, we will delve into designing the cost function and implementing the SOMA optimization algorithm. The following sub-sections will illustrate the procedure of establishing the cost function based on observations and employing SOMA to derive the most suitable solution for the cost function to find the best path for UAVs.

Some assumptions about speed, environment, and sensors are made to ensure the system’s proper operation. They play an essential role in building and designing the SOMA to solve the UAV problem.

**Speed**: It is assumed that the UAV can change direction and speed to avoid obstacles. The SOMA will consider sensor signals and the current environment and calculate the trajectory that the UAV can move in a safe and efficient direction.

**Sensors**: It is assumed that the UAV is equipped with appropriate sensors to detect and locate obstacles, other UAVs, and itself in the environment.

**Environment**: It is assumed that information about the environment surrounding the UAV is continuously updated. Spheres represent real-world obstacles.

### 3.1 Observations

Our scenario involves UAVs navigating a workspace containing hidden obstacles (detected by sensors) and corresponding targets. UAVs must avoid both obstacles and collisions with other UAVs. To formulate the problem, we consider the following key factors:

- The distance from the UAV to the target (denoted as *g*_(*X*)_) is as close as possible, guaranteeing it can catch its target;
- The distance from the UAV to the detected obstacles (denoted as *h*_(*X*)_) should be maintained as far as possible, guaranteeing avoiding collisions and ensuring safety during the movement of the UAV;
- Obstacles that are too far away do not need to be considered because they do not significantly affect the operation of the UAV.

These arguments provide essential foundations for formulating the problem and lead to Eq 1, where *f*_(*X*)_ is the objective function to be built.

$$f_{\left(X\right)}=g_{\left(X\right)}+h_{\left(-X\right)}$$ (1)

Objective function *f*_(*X*)_ is central to the trajectory planning approach. The function is designed to minimize the distance to the target while maximizing the distance from obstacles, ensuring efficient and safe navigation for the UAVs.

It combines two components. *g*_(*X*)_ represents the objective of minimizing the distance to the target. This term encourages the UAV to move towards its designated target efficiently. *h*_(−*X*)_ represents the objective of maximizing the distance from detected obstacles, including other UAVs. This term promotes safety by penalizing trajectories that bring the UAV too close to obstacles.

**Constraints**: We define the constraints governing the UAVs’ motion and how they are incorporated into the optimization problem:

- Kinematic constraints: UAVs have limitations on maximum speed, acceleration, and turning angles to ensure that planned trajectories are physically feasible. These constraints are considered during the trajectory optimization process to generate realistic flight paths.
- Safety constraints: A minimum safe distance must be maintained from obstacles and other UAVs to prevent collisions. This constraint is integrated into the cost function through the obstacle avoidance term *h*_(−*X*)_, which significantly increases the cost when the UAV approaches the safety threshold distance.
- Environmental constraints: The UAVs operate within a bounded workspace, and the environment may contain dynamic changes, such as moving obstacles. Our method assumes continuous updates of the environment information, allowing the UAVs to adapt their trajectories in real-time.

### 3.2 Analysis

To ensure the effectiveness of the cost function, we need to carefully analyze the relationship between the distance to the target and the distance to obstacles, and how they contribute to the overall cost.

**For targets**: If the cost function (*f*_(*X*)_) is linearly proportional to *g*_(*X*)_ (i.e., $f_{\left(X\right)}\propto g_{\left(X\right)}$), the value of the cost function will be big when the target is very far away and very small when the target is very close to the UAV. Meanwhile, the maximum speed of movement of the UAV is limited and does not entirely depend on the distance from the target. So, the exponential function is a suitable choice, as in Eq 2.

$$g_{\left(X\right)}=a_{1}*e^{a_{2}*dis_{tar}^{a_{3}}}$$ (2)

**Fig 1:**
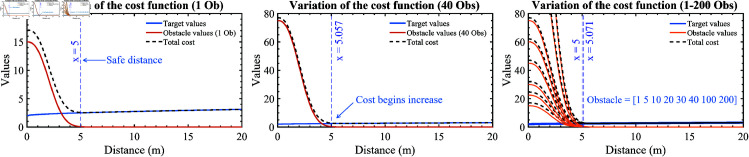
Investigate the variation of the cost function in situations where many obstacles and other robots are detected.

In which *dis*_*tar*_ is the distance from the UAV to its target, and *a*_1_, *a*_2_, and *a*_3_ are adjustment coefficients to adjust the curve (as plotted in Fig 1) and meet the specific requirements of the problem.

**For obstacles**: Similarly, the cost function value will increase significantly if the UAV is too close to obstacles, surpassing a certain given safe distance. On the contrary, obstacles will not significantly affect the cost function value if the UAV does not violate this safe distance. It can be seen if the cost function *f*_(*X*)_ is inversely linearly proportional to *h*_(−*X*)_ (i.e., $f_{\left(X\right)}\propto h_{\left(-X\right)}$ ), then the cost function value does not satisfy those conditions. In this case, the exponential function is suitable, as in Eq 3.

$$h_{\left(-X\right)}=b_{1}*\sum_{n=0}^{n_{obs}}e^{-b_{2}*dis_{obs}^{b_{3}}}$$ (3)

In which *dis*_*obs*_ is the distance from the UAV to obstacles, *b*_1_, *b*_2_, and *b*_3_ are adjustment coefficients, and *n*_*obs*_ is the number of detected obstacles.

Combining Eqs 1, 2, and 3, we have Eq 4.

$$f_{\left(X\right)}=g_{\left(X\right)}+h_{\left(-X\right)}=a_{1}*e^{a_{2}*dis_{tar}^{a_{3}}}+b_{1}*\sum_{n=0}^{n_{obs}}e^{-b_{2}*dis_{obs}^{b_{3}}}$$ (4)

All models have no specific values of the coefficients (*a*_*i*_ and *b*_*i*_, i=1,2,3). These values will be investigated and chosen depending on each designed model’s physical dimensions and the desired safe distance to obstacles. Despite the coefficient values, the algorithm performance is beyond this variation.

**Fig 2:**
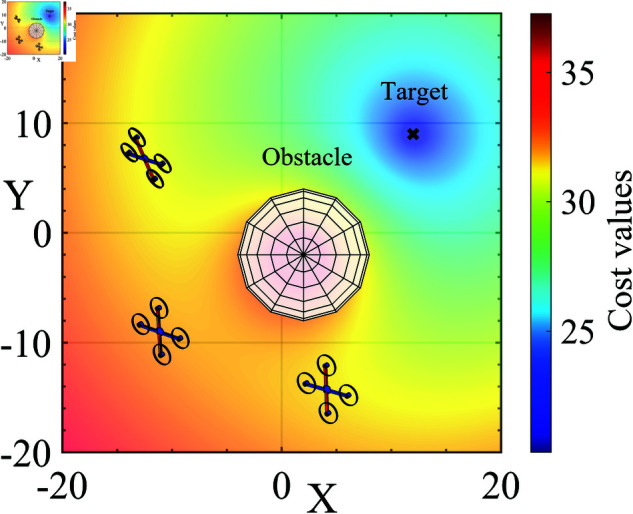
Visualization of the UAVs’ movement in the cost space.

**Visualization:** The movement of UAVs in the cost space is visualized in Fig 2. Cooler colors represent the spatial region with smaller cost values, where the UAVs will move. On the contrary, warmer colors have greater cost values. The closer to the obstacle is, the more the cost value increases, and the UAVs move away from these hot-colored areas toward cooler areas.

**Large-scale obstacle situation:** How does the optimization process scale with many more UAVs and obstacles? In a situation where many obstacles and other UAVs are detected due to the structure of the proposed cost function, they will be added to the second part of the cost function in Eq 4. Then, the total cost values will be changed. Nevertheless, the safe distance looks like not change when obstacles increase a lot. We summarized the variation of the cost function into a single plot, from 1 to 200 obstacles, as shown in Fig 1.

The cost function, as described above, provides a framework for evaluating potential UAV trajectories. To find the optimal trajectory that minimizes this cost function, we will apply the self-organizing migrating algorithm.

### 3.3 The self-organizing migrating algorithm

This study leverages the self-organizing migrating algorithm (SOMA) [12,33,34] to plan trajectories and enable obstacle avoidance for UAVs. SOMA, a swarm intelligence algorithm, draws inspiration from the cooperative-competitive behaviors observed in natural animal swarms, such as ants, bees, and bird flocks. SOMA operates by defining a population of individuals within a three-dimensional search space. These individuals are initially randomly distributed within the search space, as defined by Eq 5. Each individual represents a potential solution, which corresponds to a specific trajectory that the UAV will follow.

$${\vec{P}}_{SOMA}={\vec{x}}_{lowest}+({\vec{x}}_{highest}-{\vec{x}}_{lowest}).{\vec{r}}_{rand[0,1]}$$ (5)

where:

- ${\vec{P}}_{SOMA}$: the SOMA population,
- ${\vec{x}}_{lowest}$: the lowest boundary vector,
- ${\vec{x}}_{highest}$: the highest boundary vector,
- ${\vec{r}}_{rand[0,1]}$: random vector, in the range of [0,1].

The search process in the SOMA includes two main phases: organization and migration. During the organization phase, an individual with the smallest fitness value is chosen as the Leader, and the remaining individuals in the population are migrating individuals. All individuals will change their position based on information from the Leader and the environment.

In the migration phase, migrating individuals are selected to migrate from their current position to the Leader. They interact and cooperate with the Leader to search for potential regions by moving *step*-by-*step* toward the Leader. This migration process aims to explore and learn about novel potential regions. Migrating individuals will displace their former position based on criteria such as increasing population diversity, optimizing target values, or exploring unexplored areas. This process is performed based on Eq 6.

$${\vec{x}}_{next}^{loop+1}={\vec{x}}_{current}^{loop}+step\;({\vec{x}}_{leader}^{loop}-{\vec{x}}_{current}^{loop}).{\vec{P}}_{PRTVector}$$ (6)

where:

- ${\vec{x}}_{next}^{loop+1}$: the new position in the next loop,
- ${\vec{x}}_{current}^{loop}$: the position in the current loop,
- ${\vec{x}}_{leader}^{loop}$: the leader position in the current loop,
- *step*: the moving step of each individual,
- ${\vec{P}}_{PRTVector}$: the perturbation element vector.

During migration, individuals can exchange information with each other and receive influence from other individuals in the population, leading to achieving a balance between exploration and exploitation during the search process.

The SOMA uses a mechanism to self-adjust ${\vec{P}}_{PRTVector}$ parameters, using Eq 7. If a generated random number *rand*_*j*_ on dimension *j* is less than a given *threshold*, the value of $P_{PRTVector_{j}}$ is 1, i.e., a movement is performed in direction *j*. Otherwise, no movement takes place. These parameters change the algorithm’s search performance, allowing SOMA to adapt and optimize the moving process over time.

$$if\;rand_{j}<threshold;\;P_{PRTVector_{j}}=1;\;\;else,\;0$$ (7)

At the start of the movement process, which is also the start of the SOMA algorithm, the UAV receives signals from sensors arranged on its body to determine its position, the target position, and surrounding obstacles. Then, a population of fictitious points is created around the standing position of that UAV. These points are evaluated using the cost function in Eq 4, and the best point is selected as the Leader. The remaining positions will move towards the Leader to find the best new position on their paths. When the fictitious migration ends, the population’s new position is updated, and the first computational loop ends. Note that during this computational loop, the UAV remains in its state. The SOMA algorithm is pseudocoded as in Algotithm 1.

**Algorithm 1.** The self-organizing migrating algorithm



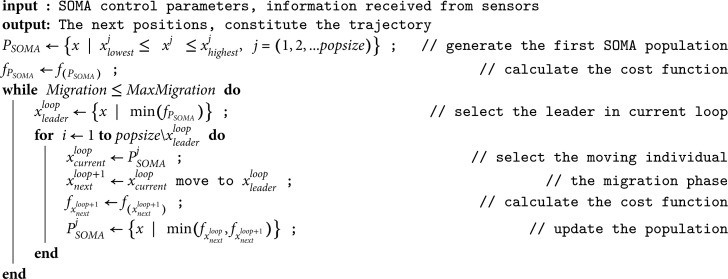



**Fig 3:**
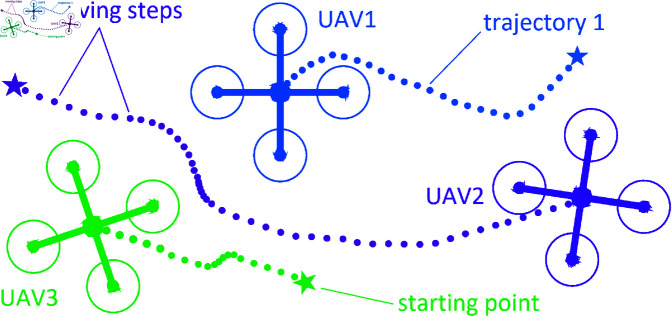
The trajectory model of UAVs with moving steps represented by dotted lines.

The searching process is repeated until a given number of migration loops is reached, and the best new position is found within the maximum allowable moving range of the UAV. Next, the UAV will move to the point it just found. And the set of points the UAV continuously finds in real time creates a trajectory that the UAV will go through, satisfying the conditions for moving, avoiding obstacles, and catching the target, as shown in Fig 3.

## 4 Experimental setup

### 4.1 Simulation scenarios

Three test scenarios were designed to evaluate the feasibility and effectiveness of the proposed method, covering actual conditions commonly encountered during UAV operation.

Two main aspects are considered in the test scenarios: The UAV’s ability to avoid obstacles and reach targets in an unknown environment.

**Fig 4:**
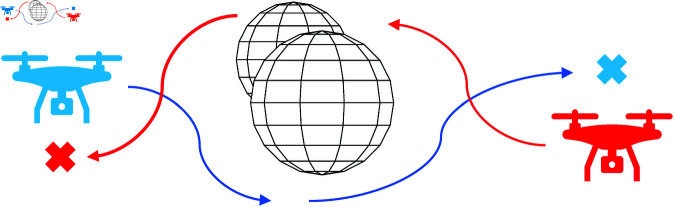
The first movement scenario contains two UAVs and two obstacles.

In the first scenario, we consider the simplest case with two UAVs moving in a space containing two fixed obstacles. UAVs and obstacles are intentionally placed to create obstructions, forcing UAVs to avoid them and each other, as shown in Fig 4.

**Fig 5:**
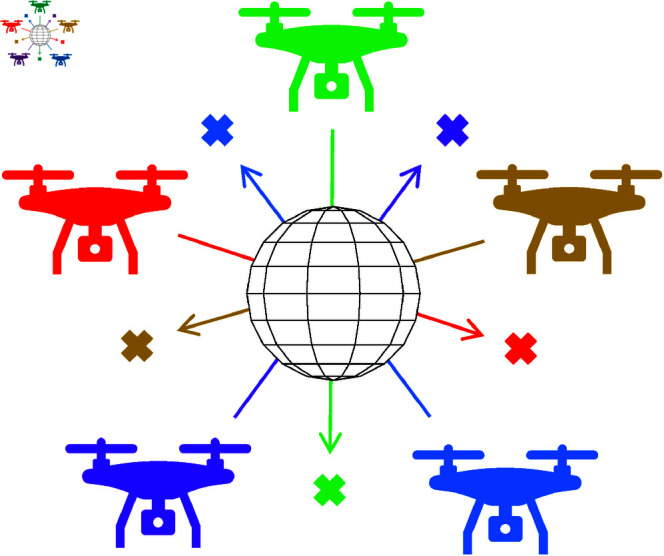
The second movement scenario includes five UAVs and one obstacle.

The second scenario includes five UAVs and a static obstacle. The UAVs are positioned so that they become dynamic obstacles for each other, as shown in Fig 5.

**Fig 6:**
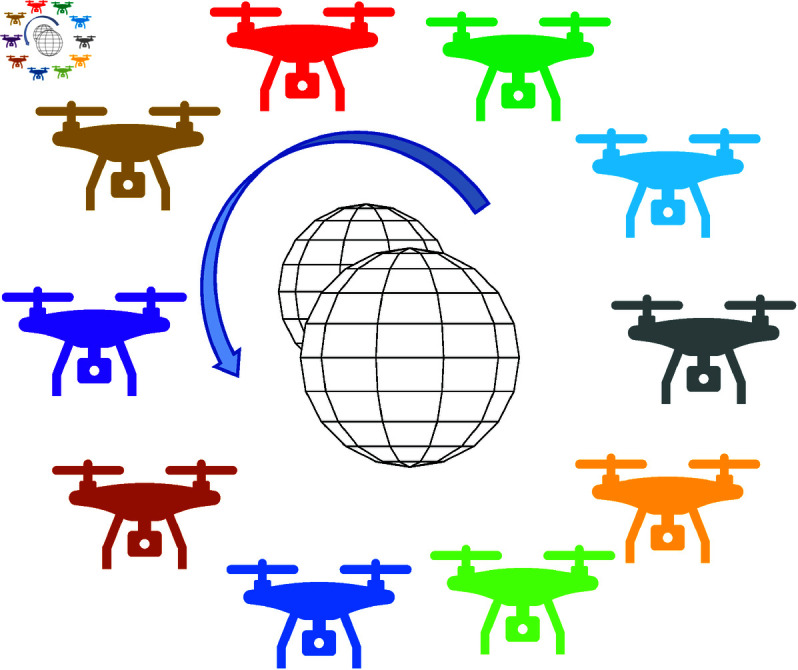
The third movement scenario includes ten UAVs and two obstacles.

In the third scenario, we consider a space with ten UAVs and two static obstacles. The UAVs and obstacles are arranged at different positions and heights to test the comprehensive obstacle avoidance ability of the UAVs, as shown in Fig 6. The locations of UAVs and obstacles are given in Table 1. The safe distance for all scenarios is set at 5*m*.

**Table 1:** Arrangements of UAVs and obstacles in Cartesian space (in meter).

Scenario	Objects	$x_{start}$	$y_{start}$	$z_{start}$	$x_{stop}$	$y_{stop}$	$z_{stop}$	radius
1	UAV1	–8	–6	–1	8	1	–1	1.2
1	UAV2	8	6	2	–8	–3	2	1.2
1	OB1	2	–4	–1	—	—	—	5
1	OB2	0	4	2	—	—	—	4
2	UAV1	0	11	–1	0	–11	1	1.2
2	UAV2	11	6	1	–8	–9	–1	1.2
2	UAV3	11	–8	1	–11	8	1	1.2
2	UAV4	–11	–7	–1	9	9	1	1.2
2	UAV5	–8	10	1	8	–10	1	1.2
2	OB	0	0	0	—	—	—	5
3	UAV1	10	9	–7	–5	–14	6	1.2
3	UAV2	2	–12	–6	–1	14	10	1.2
3	UAV3	–11	–12	–7	9	16	8	1.2
3	UAV4	–12	12	–10	8	–13	7	1.2
3	UAV5	12	–3	7	–16	1	6	1.2
3	UAV6	–6	8	–8	16	4	5	1.2
3	UAV7	13	13	7	13	3	–7	1.2
3	UAV8	13	–13	6	–11	8	–7	1.2
3	UAV9	–9	14	7	–13	–3	–8	1.2
3	UAV10	–16	–14	6	6	–5	–7	1.2
3	OB1	–3	–5	5	—	—	—	4
3	OB2	1	9	–1	—	—	—	3

### 4.2 Parameters

The parameters of the SOMA are given in Table 2. These parameters are set according to recommendations from the original publication [12].

**Table 2:** Control parameters of the SOMA algorithm.

step	PathLength	threshold	Max Migration	Population Size
0.11	3	0.3	20	50

Our tests are performed on the personal computer using the $13^{\text{th}}$ Gen Intel(R) Core(TM) i7-13700KF CPU, base speed of 3.40 GHz, and 64 GB RAM. The operating system used is Windows 11 Pro 64-bit 23H2, with Matlab R2023b version.

## 5 Results and discussion

### 5.1 Solution effectiveness

To consider the effectiveness of the cost function we built and the performance of the obstacle avoidance and catching target for UAVs using the SOMA algorithm, we have gathered simulation data from three scenarios that have been designed. For each scenario, we analyzed the two critical metrics to evaluate the effectiveness of the proposed method:

- **Collision rate** (%): Assesses the rate of collisions with obstacles during flight, including collisions with other UAVs. This metric indicates the algorithm’s ability to avoid collisions and maintain safety for UAVs;
- **Target reachability** (Reached/Not,%): Assess the ability of UAVs to approach a given target. We consider whether the UAVs can reach the target, and if not, will the distance to the target decrease or increase? This metric reflects the algorithm’s ability to orient and track its target.

To present the results visually and in detail, we recorded the entire process of moving, avoiding obstacles, and catching the target of UAVs in the form of top-view figures in the third angle (2D figures) and axonometric views (3D figures). They were taken at different times to describe the moving process most completely.

**Fig 7:**
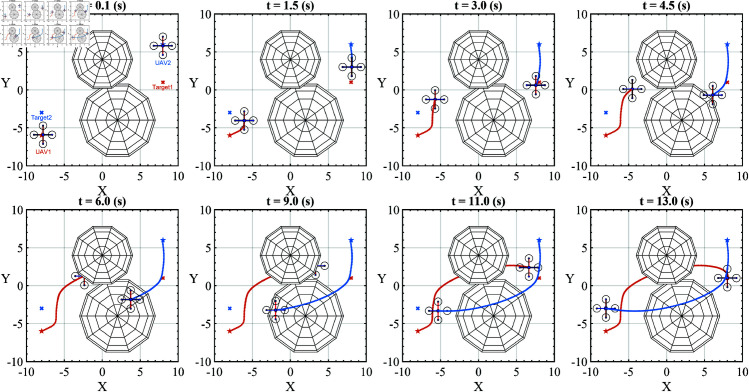
The moving process of two UAVs in the first scenario is presented from a top-view perspective.

Fig 7 shows the movement of the UAVs in the first scenario. In this experiment, we positioned only two UAVs, and two obstacles were placed between them to prevent straight movement. Each UAV operates based on an independent SOMA algorithm.

In Fig 7, the red dotted line shows the trajectory of UAV1, and the blue dotted line shows the trajectory of UAV2. At the t=0.1(s) frame, the UAVs have taken their first steps, heading towards their target. In frames from t=0.1−1.5(s), UAV1 turns in another direction because it has detected an obstacle ahead, while UAV2 moves sideways to avoid the remaining obstacle. The t=3.0−11.0(s) frames show the movement of the UAVs with continuously changing trajectories. Then, the movement continues until both UAVs reach the target at t=13.0(s).

In the first scenario, the measured simulation data show:

- Collision rate: 0%, no collision occurred (both UAV1 and UAV2);
- Target reachability: 100%, reached all (both UAV1 and UAV2).

**Fig 8:**
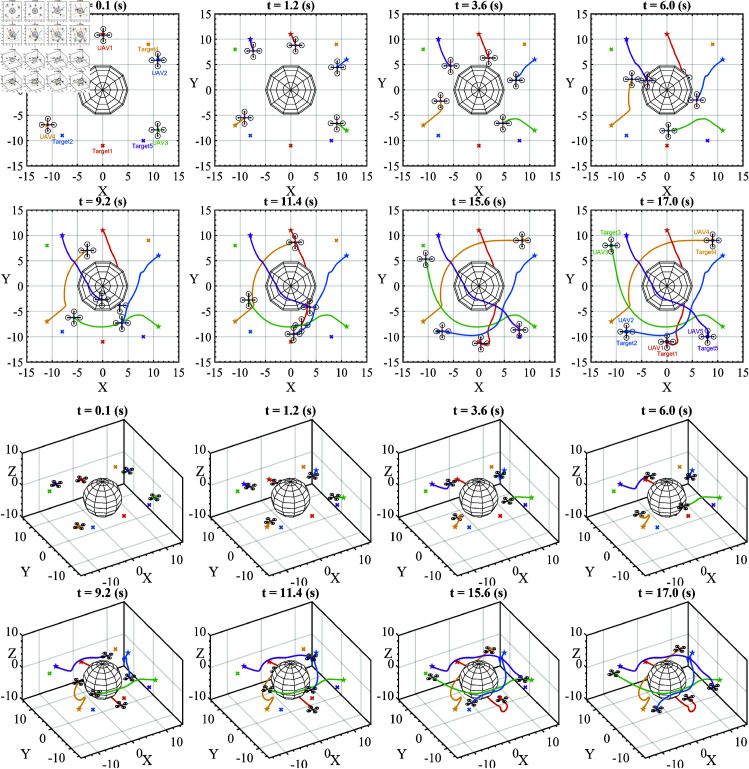
The moving process of five UAVs in the second scenario is presented from a top-view perspective of 2D figures and axonometric projection of 3D figures.

In a more complex scenario, five UAVs are placed around an static obstacle, and their targets are placed on the opposite side to increase the difficulty of movement. At this scene, the UAVs will be dynamic obstacles to each other. We can observe the movement of five UAVs in Fig 8. The red, blue, green, orange, and purple dotted lines represent the trajectories of UAVs from 1 to 5, respectively. Each UAV has a unique target, indicated by corresponding colored crosses.

Within the limit of the article’s length, the movement process of the UAVs is presented in eight frames in 2D and eight in 3D, taken at the same time, respectively.

At frame t=0.1(s), all UAVs start moving the first steps from their initial position toward their target. During movement, UAVs must change their trajectory to avoid collisions with other UAVs and static obstacles when detected. The frames from *t* = 1.2 − 15.6(s) show the continuous change in the UAV’s trajectory. Finally, all UAVs have achieved their targets at frame t=17.0(s).

The results collected from the simulation process in this scenario show:

- Collision rate: 0%, no collision occurred for any of them;
- Target reachability: 100%, five UAVs reached their target.

**Fig 9:**
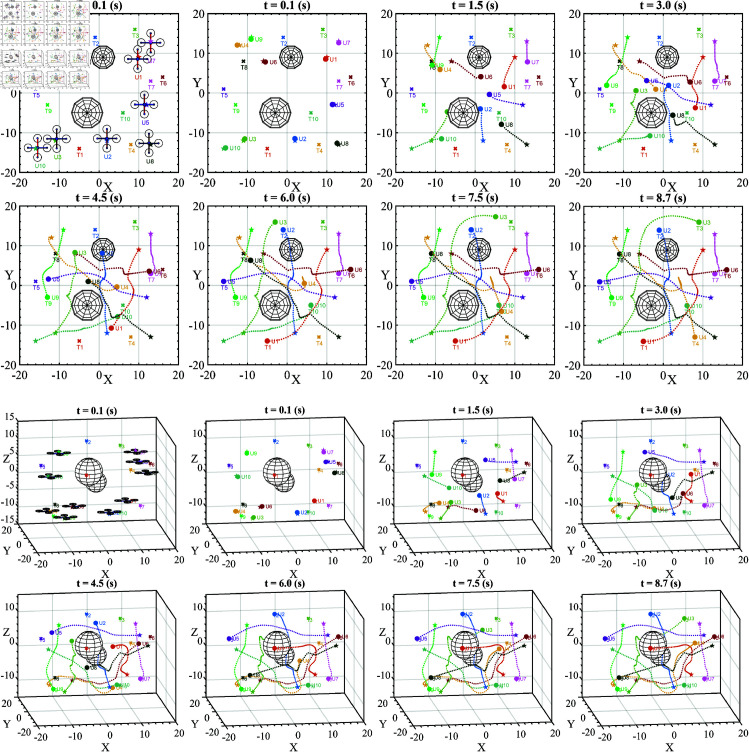
The moving process of ten UAVs and two obstacles in the last scenario is presented from a top-view perspective of 2D figures and axonometric projection of 3D figures.

In the last scenario, we placed ten UAVs and two static obstacles to simulate the most complex situation in this study. Dynamic obstacles are not necessarily because the UAVs are dynamic obstacles to each other. The targets of the UAVs are intentionally set in opposite positions (the other side of obstacles) in tight spaces. The aim is to comprehensively evaluate the ability of the proposed solution in avoiding obstacles and catching targets in a multi-UAV operating environment. We can observe the movement of ten UAVs in Fig 9.

The results obtained from the third scenario show that the collision rate and target reachability are 0% and 100%, respectively. No collision occurs between any pair of UAVs, and all UAVs reach their target.

### 5.2 Statistical analysis

To eliminate luck, increase reliability, and diversify the situations that UAVs encounter in reality, in the third scenario setup, we let the UAVs move continuously from the starting point to the target and then return to the starting point instead of just one time. In other words, when a UAV reaches the target (forward), it will return to the starting point (backward). After getting the starting point, it will continue to move towards the target, and this process will repeat for an extended period.

Due to the travel distances of each UAV to its target being different within the same period, the counting of each UAV reaching its target will be different, as well as the forward and backward trajectories. The process repeats with t=1000(s) setting time. A small piece of the simulation with t=10(s) is also presented for comfortable viewing. As expressed below, we conducted advanced analyses of distances to survey the ability to avoid obstacles and reach the target.

**Fig 10:**
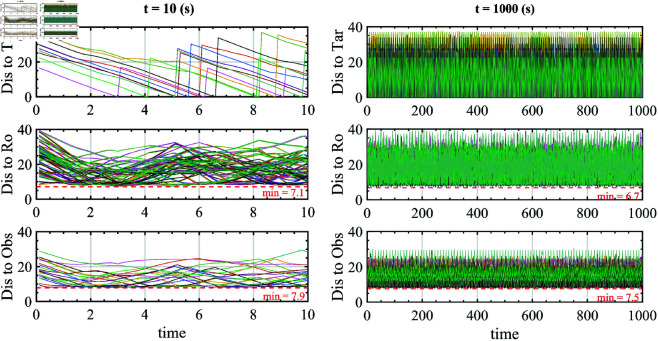
Different distances are plotted to consider the collision rate and target reachability of UAVs in the environment.

We measured and plotted different types of distances from the UAV to the rest of the environment in *meter*, as shown in Fig 10. These include the distance from each UAV to its target (denoted by Dis to Tar in Fig 10), from each UAV to obstacles (denoted by Dis to Obs), and from each UAV to the remaining nine UAVs (denoted by Dis to Ro).

For targets, Dis to Tar approaches zero, which means the UAV is gradually catching the target. When the UAV reaches the target, the starting point and the target will be swapped, so the distance is reset. It is reflected in Fig 10, and all UAVs reached their given targets. For obstacles and other UAVs, Dis to Obs and Dis to Ro will change over time but must not exceed a given safe distance. Fig 10 shows that the minimum distance value is 6.7*m*, meaning no collision occurs.

**Fig 11:**
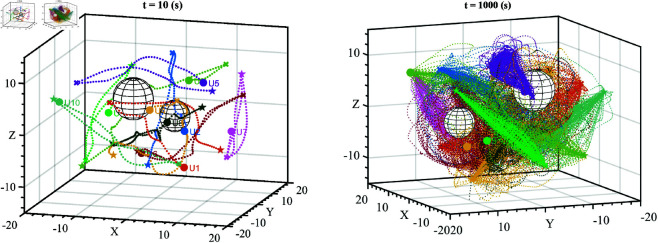
A 3D axonometric projection of the whole movement of ten UAVs and two obstacles in the repetitive test environment.

Fig 11 depicts the entire movement process of ten UAVs and two obstacles in the test environment as 3D axonometric projection, with the simulation time of t=10(s) and t=1000(s). Corresponding color dots have replaced the shape of UAVs to avoid unnecessary masking.

Fig 11 shows that the UAV’s trajectories changed and did not repeat the previous trajectory when the UAV moved toward the target or backward to the starting point. UAVs have different paths to fly to the target. Consequently, the time to reach the target also differs, leading to diverse trajectories. Then, the UAVs face obstacles and other UAVs in various situations, ensuring diverse handling situations in movement. Finally, the measurement data in Fig 10 shows that the UAVs completed the task of reaching their target without any collisions.

Through collecting and analyzing simulation data from three different scenarios, we have evaluated the correctness of the proposed cost function and the effectiveness of the SOMA algorithm in solving the problem of avoiding obstacles and catching targets for UAVs. The results show that the proposed solution achieves 0% collision rate and reliable target reachability approach in all three simulation scenarios. It proves the capability and effectiveness of the proposed method in navigating UAVs to avoid collisions and catch targets safely and accurately.

## 6 Conclusion

In this article, we have applied the self-organizing migrating algorithm to solve the problem of trajectory planning for unmanned aerial vehicles, including the problem of avoiding obstacles and catching targets in real-time. We have proposed a method to construct the cost function and apply SOMA to find the optimal solution to navigate UAVs in complex environments.

Three simulation scenarios have been set up to demonstrate correctness and effectiveness, covering most situations that UAVs face in practice. Collected data in the most complex situations repeated over a long period have also confirmed the stability of the solution.

Simulation results have shown that SOMA is an effective and flexible solution for avoiding obstacles and generating flight paths for UAVs. This algorithm allows UAVs to operate independently without depending on a central controller. SOMA is also scalable and reliable in large-scale scenarios as the number of UAVs increases.

As a practical application, we are planning further to apply our approach to drone competition like the International Unmanned Aerial Vehicle Design Competition at Ton Duc Thang University (TDTU) and the International Aerial Robotics Competition (IARC). These competitions present complex challenges that require advanced autonomous navigation, real-time obstacle avoidance, and efficient decision-making under dynamic conditions. By implementing our proposed algorithm in drones participating in these events, we aim to demonstrate its effectiveness in real-world scenarios. This will not only validate the robustness and scalability of our approach but also contribute to advancing autonomous drone technology in competitive and high-stakes environments.

This research has contributed a swarm intelligence-based approach to the trajectory planning for UAVs. Our solution provides a basis for future development and improvement of UAV systems. It can be applied in many applications, from environmental monitoring to smart agriculture and search and rescue.
